# Supplementary material for: COVID-19 Vaccination Timing, Relative to Acute COVID-19, and Subsequent Risk of Long COVID
Source: medRxiv. 2025 Apr 23:2025.04.22.25326224. Preprint. [Version 1] doi: 10.1101/2025.04.22.25326224 (PMC12045423; doi:10.1101/2025.04.22.25326224)
Supplement: Supplement 3 [file media-3.pdf]

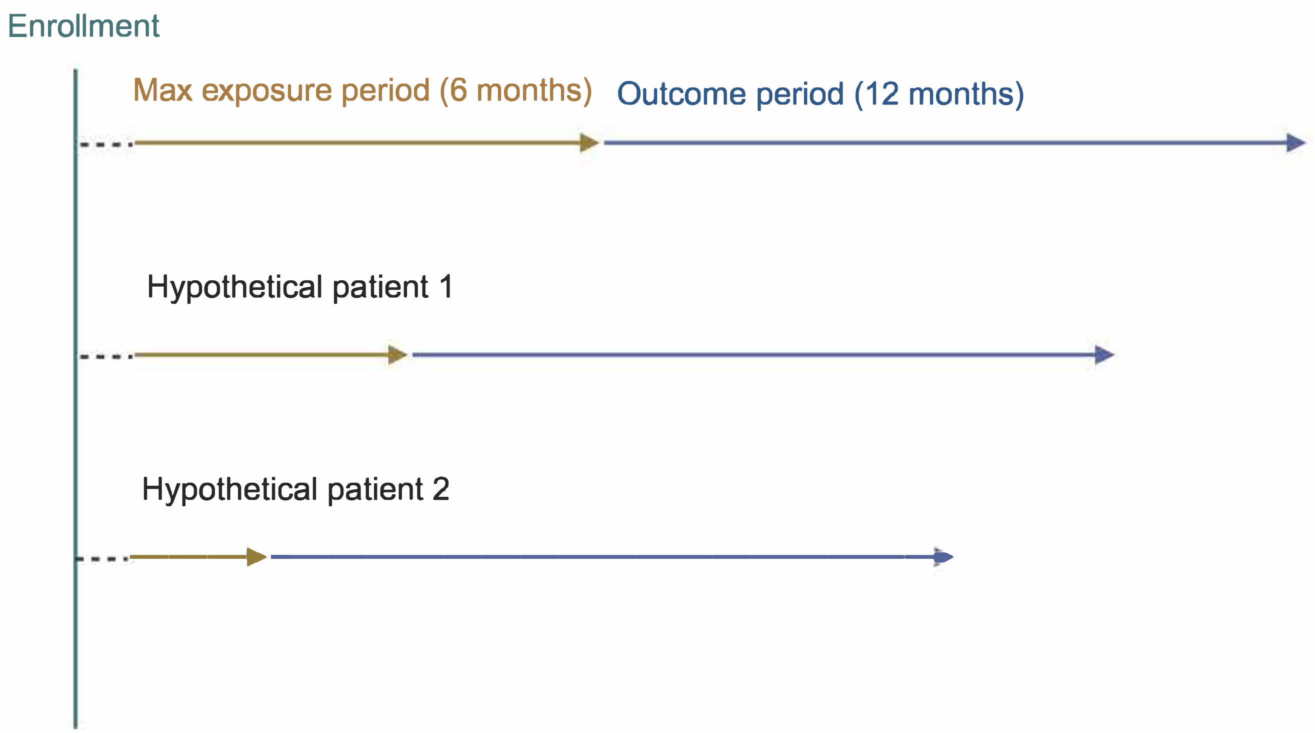

Enrollment: initial COVID-19 vaccination between December 25, 2021 and September 25, 2022. We adjusted for baseline patient variables at this point (see Methods).

Exposure period: we assessed patient acute COVID-19 status months 1-7 after enrollment. We excluded patients with COVID-19 diagnoses within 1 month of vaccination. We discretized over 2 month intervals and assessed healthcare utilization and additional vaccination doses in each interval

Outcome period: We assessed patient Long COVID status in the 12 months following acute COVID-19. The outcome period began if/when the patient was diagnosed with acute COVID-19. We discretized over 2 months intervals and assessed healthcare utilization and additional vaccination doses in each interval.
